# Graduate Education and Institutions

**Published:** 1921-08-27

**Authors:** 


### GRADUATE EDUCATION AND INSTITUTIONS.

**London School of Clinical Medicine (Post-Graduate).**—This School, which is devoted exclusively to post-graduate study, has its headquarters at the Seamen's Hospital, Greenwich. It has not been found possible to reopen this School for the full post-graduate courses held there prior to the war. There are, however, special facilities for courses in operative surgery, which are held at frequent intervals by the surgeons to the hospital. In addition, arrangements are made for utilising the unusual opportunities for the study of pathology which the Seamen's Hospital affords. Professor R. Tanner Hewlett is Director of Pathology, and Dr. Arthur Davies Assistant Director of Pathology, Dr. Frank Standish, Pathologist. Particulars of courses of study may be obtained on application to the Dean, Dr. H. Ridley Prentice.

**North-East London Post-Graduate College.**—This School, which is in connection with the Prince of Wales's General Hospital, Tottenham, N., is recognised by the University of London, the Admiralty, and the India Office, and by the University of Cambridge in regard to bacteriology for the D.P.H. diploma, as a place of post-graduate study. Opportunities are here afforded for attending demonstrations on various branches of medicine, surgery, and gynæcology, diseases of the eye, ear, throat, nose, skin, fevers, diseases of children, psychological medicine, anæsthetics, *x*-rays, bacteriology, and dentistry. Further details as to fees and special courses of teaching may be obtained from the Dean at the hospital.

**West London Post-Graduate College.**—West London Hospital, Hammersmith. The hospital itself contains 160 beds, and the advantages of the direct connection of the College with the wards and out-patient work are obvious. It is recognised by the Royal College of Surgeons as an institution where candidates (who need not be members) for the Fellowship can spend the necessary year of study after obtaining a registrable qualification, while the College and hospital are also recognised by the naval and military authorities as regards study leave. The hospital is authorised by the University of London to give certificates of post-graduate study for the M.D. and M.S. degrees. Members of the College may also accompany the resident staff on their visit to the wards. Operations are performed daily at 2.30 P.M. Post-graduates are allowed to stand near the table and can see the operations perfectly. The surgeons often avail themselves of the assistance of post-graduates. During the sessions lectures are given daily, except Saturdays, at 5 P.M., on practical medicine and surgery in all their branches. There are also short courses on tropical and mental diseases by specialists in those subjects. Small classes are formed, when required, in connection with diseases of special regions. For these classes, each of which is strictly limited to a small number, additional fees are required, as they are practically individual and tutorial. The ordinary fee for membership covers attendance at all general lectures and at all the routine practice of the hospital, whether in the general or special wards or departments. Autopsies can, of course, be attended. Arrangements can be made through the College authorities for special coaching should it be desired. The V.D. Department is open daily, Saturdays included, at 5.30 P.M. It is one of the largest in London. Members may join for any period from one week upwards, the fees ranging from £1 11s. 6d. to £45.

**London School of Tropical Medicine, University of London.**—The curriculum is so arranged as to equip graduates to sit for the D.T.M.L.H. Cambridge and the D.T.M. of the London Conjoint Board. There are three sessions annually, commencing September 19, January 15, and May 1. The ordinary course extends over a period of twelve weeks. There are special departments in entomology, helminthology, and protozoology. There is a large library and museum, and the school is provided with the latest scientific requirements. About 200 students attend the various classes annually. Special facilities for research are provided, and there are two scholarships in the gift of the school. The school premises are situate in the same building as the Hospital for Tropical Diseases, Endsleigh Gardens, Euston Road, N.W., one of the hospitals of the Seamen's Hospital Society, at which tropical diseases only are treated. Particulars may be obtained from the Dean, Sir Havelock Charles, G.C.V.O., at the India Office, or from the Secretary at the Head Office, Seamen's Hospital, Greenwich.

**Liverpool School of Tropical Medicine.**—The Liverpool School of Tropical Medicine is carried on in conjunction with the University of Liverpool and the Royal Infirmary, for the purposes of research and clinical teaching respectively. A special ward at the hospital has been set apart for the reception and treatment of tropical diseases, and facilities for investigation and study are given at the new laboratories of the school at the University. The next courses of instruction for the Diploma in Tropical Medicine, each lasting about three months, will begin on or about September 15, 1921, and January 6, 1922.

**Both Cambridge University and the English Conjoint Board** hold examinations for the Diploma in Tropical Medicine. Neither of these bodies teaches Tropical Medicine; candidates derive their knowledge of the subject elsewhere, usually at either the London or the Liverpool School. Diplomas are granted by Cambridge in Sanitary Science, Psychological Medicine, and Medical Radiology and Electrology. Teaching is provided for all three Examinations.

**Edinburgh Diploma of Tropical Medicine.**—The University of Edinburgh grants a Diploma in Tropical Medicine and Hygiene. The examinations are held in December and June. The fees for the first and any subsequent appearance are: Bacteriology, £1 1s.; diseases of tropical climates, £1 1s.; tropical hygiene, £1 1s.; tropical clinical medicine, £1 1s.; medical entomology and parasitology, £1 1s.; total, £5 5s.

